# Gut archaea associated with bacteria colonization and succession during piglet weaning transitions

**DOI:** 10.1186/s12917-022-03330-4

**Published:** 2022-06-24

**Authors:** Xinwei Xiong, Yousheng Rao, Xutang Tu, Zhangfeng Wang, Jishang Gong, Yanbei Yang, Haobin Wu, Xianxian Liu

**Affiliations:** 1 Institute of Biological Technology, Nanchang Normal University, Nanchang, Jiangxi 330032 People’s Republic of China; 2 Key Laboratory of Women’s Reproductive Health of Jiangxi, Jiangxi Provincial Maternal and Child Health Hospital, Nanchang, Jiangxi 330006 People’s Republic of China

**Keywords:** Piglet, Metagenomic sequencing, Gut microbiota, Archaea, Eukaryotes, Weaning

## Abstract

**Background:**

Host-associated gut microbial communities are key players in shaping the fitness and health of animals. However, most current studies have focused on the gut bacteria, neglecting important gut fungal and archaeal components of these communities. Here, we investigated the gut fungi and archaea community composition in Large White piglets using shotgun metagenomic sequencing, and systematically evaluated how community composition association with gut microbiome, functional capacity, and serum metabolites varied across three weaning periods.

**Results:**

We found that Mucoromycota, Ascomycota and Basidiomycota were the most common fungi phyla and Euryarchaeota was the most common archaea phyla across individuals. We identified that *Methanosarcina siciliae* was the most significantly different archaea species among three weaning periods, while *Parasitella parasitica*, the only differential fungi species, was significantly and positively correlated with *Methanosarcina siciliae* enriched in day 28 group. The random forest analysis also identified *Methanosarcina siciliae* and *Parasitella parasitica* as weaning-biased archaea and fungi at the species level. Additionally, *Methanosarcina siciliae* was significantly correlated with *P. copri* and the shifts of functional capacities of the gut microbiome and several CAZymes in day 28 group. Furthermore, characteristic successional alterations in gut archaea, fungi, bacteria, and serum metabolites with each weaning step revealed a weaning transition coexpression network, e.g., *Methanosarcina siciliae* and *P. copri* were positively and significantly correlated with 15-HEPE, 8-O-Methyloblongine, and Troxilin B3.

**Conclusion:**

Our findings provide a deep insight into the interactions among gut archaea, fungi, bacteria, and serum metabolites and will present a theoretical framework for understanding gut bacterial colonization and succession association with archaea during piglet weaning transitions.

**Supplementary Information:**

The online version contains supplementary material available at 10.1186/s12917-022-03330-4.

## Background

The gut microbiome is a complex ecosystem comprised of interacting communities of bacteria, fungi, viruses, and archaea [1]. There is a great deal of evidence, for instance, that the intestinal microbiome plays crucial roles in pig health, and is linked with daily growth and body weight [2], nutrient processing and energy harvesting [3], and fat storage [4]. However, the majority of these findings attributed changes in porcine health to bacteria, which are not the only components of gut microbiomes. To date, very little work has been done on the role of the fungal and archaeal microbiota components.

There are over 3.8 million species of fungi, which display an immense diversity of life forms, nutritional strategies, and associations with other organisms [5, 6]. For example, infections by parasitic eukaryotes result in decreased allergic and autoimmune disease prevalence [7] and have been used for therapeutic interventions in that context [8, 9]. The fungus *Candida* can decompose starch (i.e., carbohydrates in foods) into monosaccharides, providing the raw materials for bacterial fermentation [10]. Furthermore, recent studies have demonstrated that some fungi, like *Candida albicans*, can cause disease under the right circumstances, including failure to thrive [11, 12]. However, we still know little about the role that eukaryotes play within the host microbiome and their impacts on the host health.

Archaea are single-celled prokaryotes with distinct cellular characteristics that separate them from bacteria and eukaryotes, such as lack of peptidoglycan and D-glycerol esters or fatty acids [13]. Some archaeal species are mesophilic [14] and others are stable commensals of the gastrointestinal tract where they perform functions such as methanogenesis, transformation of heavy metals, trimethylamine metabolism, and immune modulation [15–18]. The most common gut archaea is *Methanobrevibacter smithii* [19, 20], an obligate carbon dioxide (CO_2_) reducing species that produces methane from side products of bacterial fermentation [21]. Methanogens drive effective degradation of organic substances and consequentially, *M. smithii* plays a key role in interspecies hydrogen transfer by maintaining syntrophic relationships with bacterial populations [22].

In piglets, the process of weaning presents a unique challenge to porcine gut physiology [23]. To date, only a few studies have examined the development of the gut fungi and archaea during the suckling and weaning periods [12, 24–26]. However, relatively little is known about the gut microbiome interactions (e.g. fungi-archaea interactions) and host-microbial interactions (e.g. archaea-serum metabolites interactions) during the weaning transition. In this study, we collected the feces samples from piglets at three different ages, 14, 21, and 28 days during weaning periods. Then, shotgun metagenomic sequencing was performed on fecal samples to comprehensively characterize porcine gut fungi and archaea composition during weaning periods. Furthermore, we constructed a co-occurrence network based on the relative abundances of differential gut archaea, fungi and bacteria species and serum metabolites during the three weaning periods to further uncover how these signatures modulated host metabolism.

## Results

### The taxonomic characterization of swine gut fungi and archaea

The gut fungi and archaea of five Large White piglets (two males and three females) across three age strata were studied. Fresh fecal sampling continued for all five piglets at 14 days (day 14 group), 21 days (day 21 group, the day of weaning), and 28 days (day 28 group) of age. To taxonomically characterize the swine gut fungi and archaea, we performed shotgun metagenomic sequencing on all 15 fecal samples. In total, we generated ~ 3,232 million base-pairs (bp). A sequence assembly analysis of the samples produced 1,566,160 contigs with an average length of 2,090 bp and an average N50 and N90 length of 4,599 bp and 707 bp, respectively (Supplementary Table 1). We determined the phylogenetic composition of the fecal microbiota by blasting against the National Center for Biotechnology Information (NCBI) non-redundant (NR) database. The results showed that 98.46% non-redundant genes were assigned to the bacteria super kingdom, whereas 0.58% genes, 0.05% genes and 0.91% genes were annotated as viruses, eukaryotes and archaea, respectively.

**Fig. 1:**
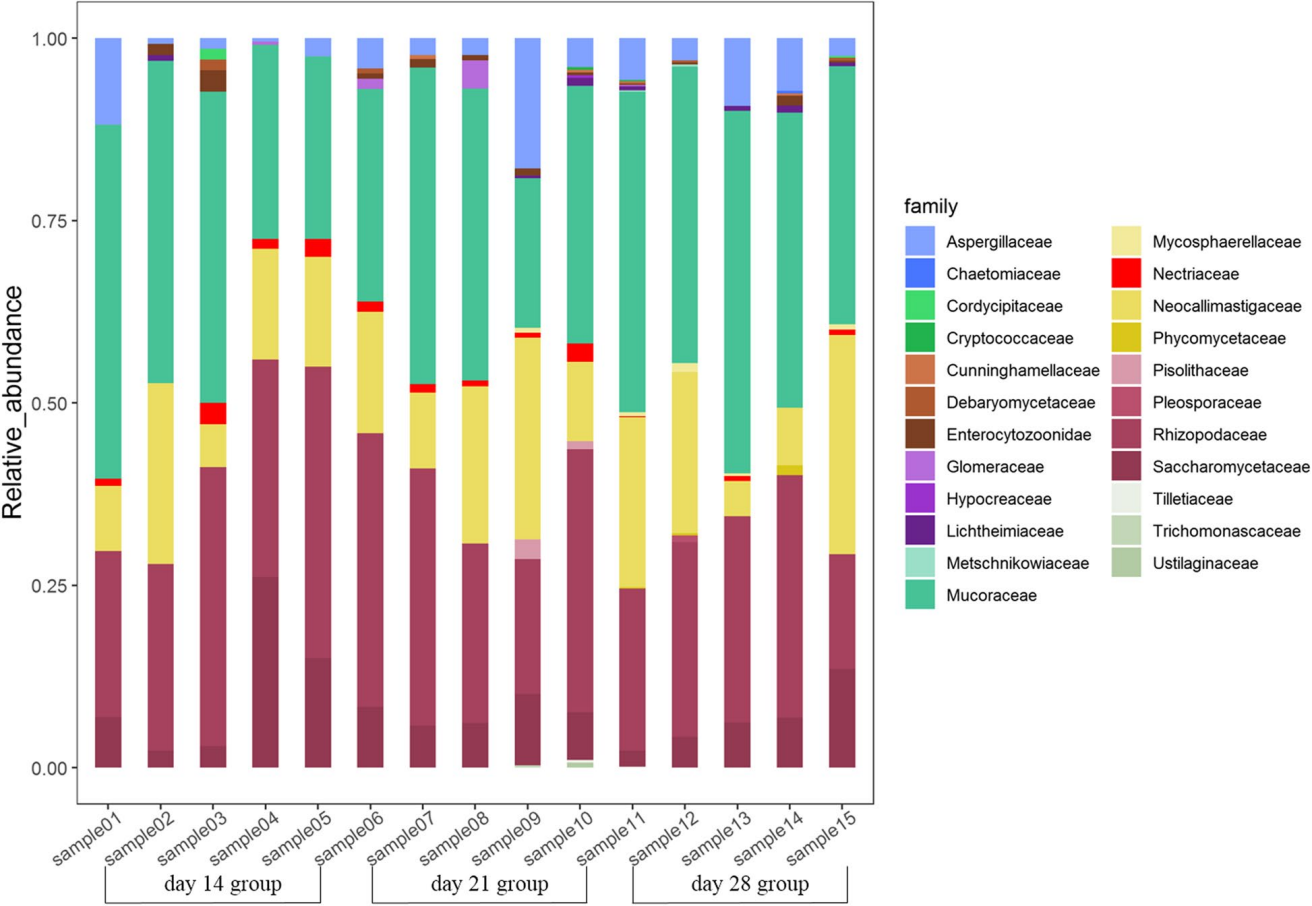
Categories and relative abundance of gut fungi at the family level by shotgun metagenomic sequencing data for all tested samples

**Fig. 2:**
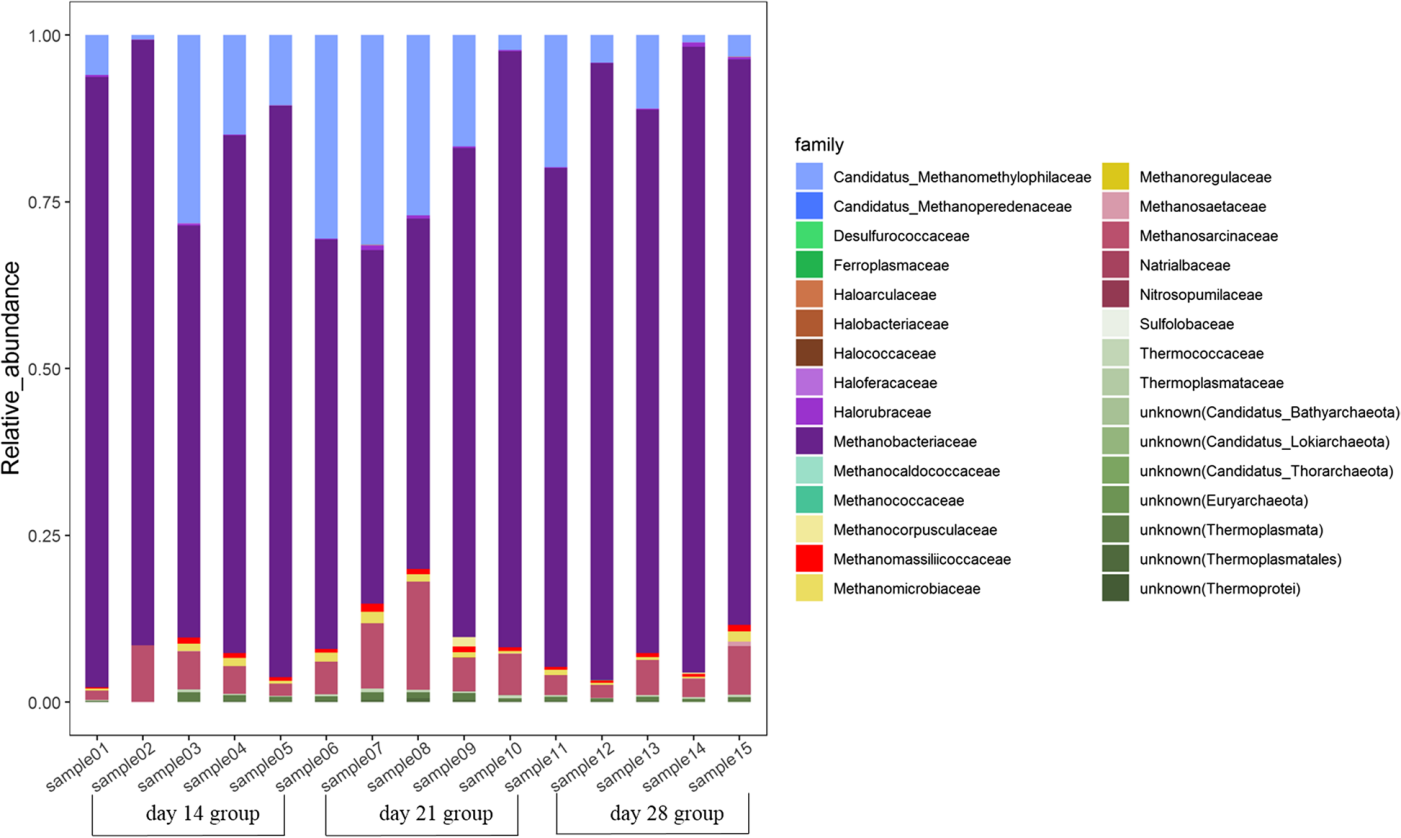
Categories and relative abundance of gut archaea at the family level by shotgun metagenomic sequencing data for all tested samples

We then identified the taxonomic composition of swine gut fungi and archaea at the phylum, family and species levels. The Mucoromycota (62.94%—97.56%), Ascomycota (0.89%—31.06%) and Basidiomycota (1.30%—7.63%) were the most common fungal phyla present across individuals (Supplementary Fig. 1A). The top 3 most common fungal families were Mucoraceae, Rhizopodaceae, and Neocallimastigaceae, with an average abundance across samples of 37.68%, 28.96% and 16.33%, respectively (Fig. 1). A total of 23 species were found to be present across all 15 samples, with *Parasitella parasitica* being the most abundant fungi species (Supplementary Fig. 2A). For archaea, we identified 6 phyla, 30 families and 36 species present in all 15 samples. Euryarchaeota was the most abundance phylum (Supplementary Fig. 1B), and Methanobacteriaceae, Methanosarcinaceae, and Candidatus Methanomethylophilaceae were the most abundant families (Fig. 2). Moreover, *Methanobrevibacter smithii* was the most abundance species, followed by *Candidatus Methanomethylophilus alvus* (Supplementary Fig. 2B).

### Variations in the gut fungi and archaea community across three weaning periods

**Fig. 3:**
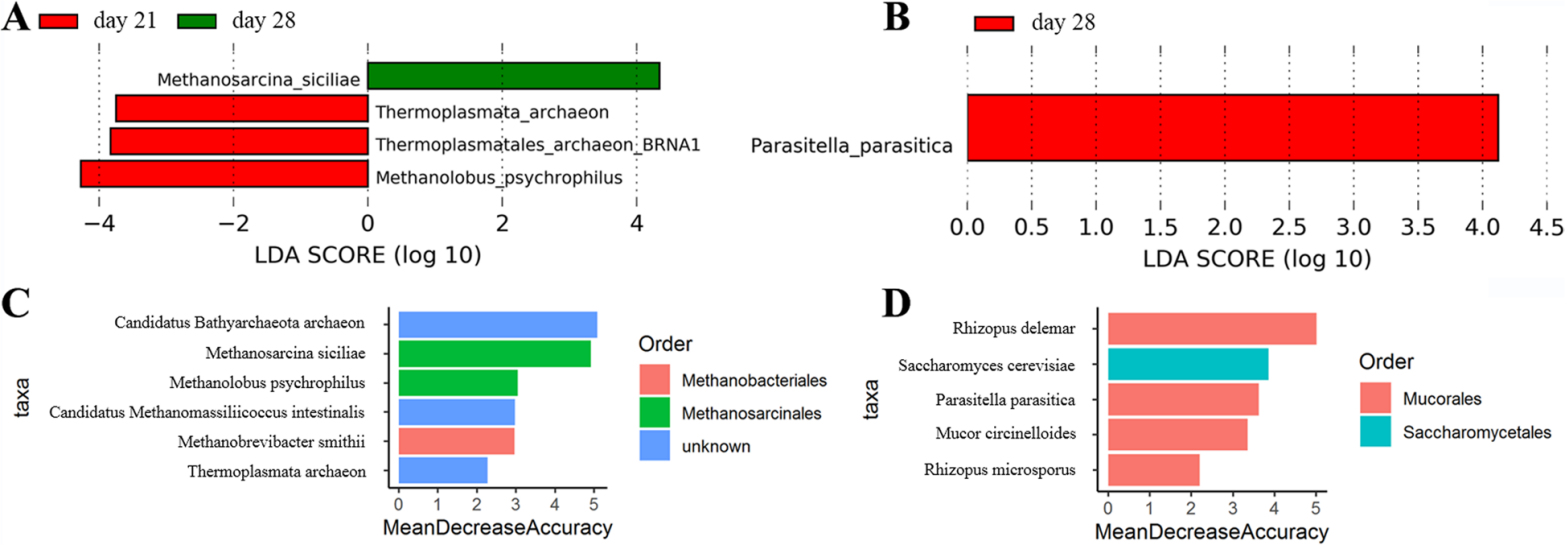
Changes in gut archaea and fungi composition based on metagenomic sequencing results across the three weaning periods. (**A**) Differences between gut archaea species. LDA score ≥ 2 was set as the threshold. (**B**) Differences between gut fungi species. LDA score ≥ 2 was set as the threshold. (**C**) Discrimination of samples from different weaning periods based on the gut archaea species level from a Random Forest analysis. (**D**) Discrimination of samples from different weaning periods based on the gut fungi species level from a Random Forest analysis

**Fig. 4:**
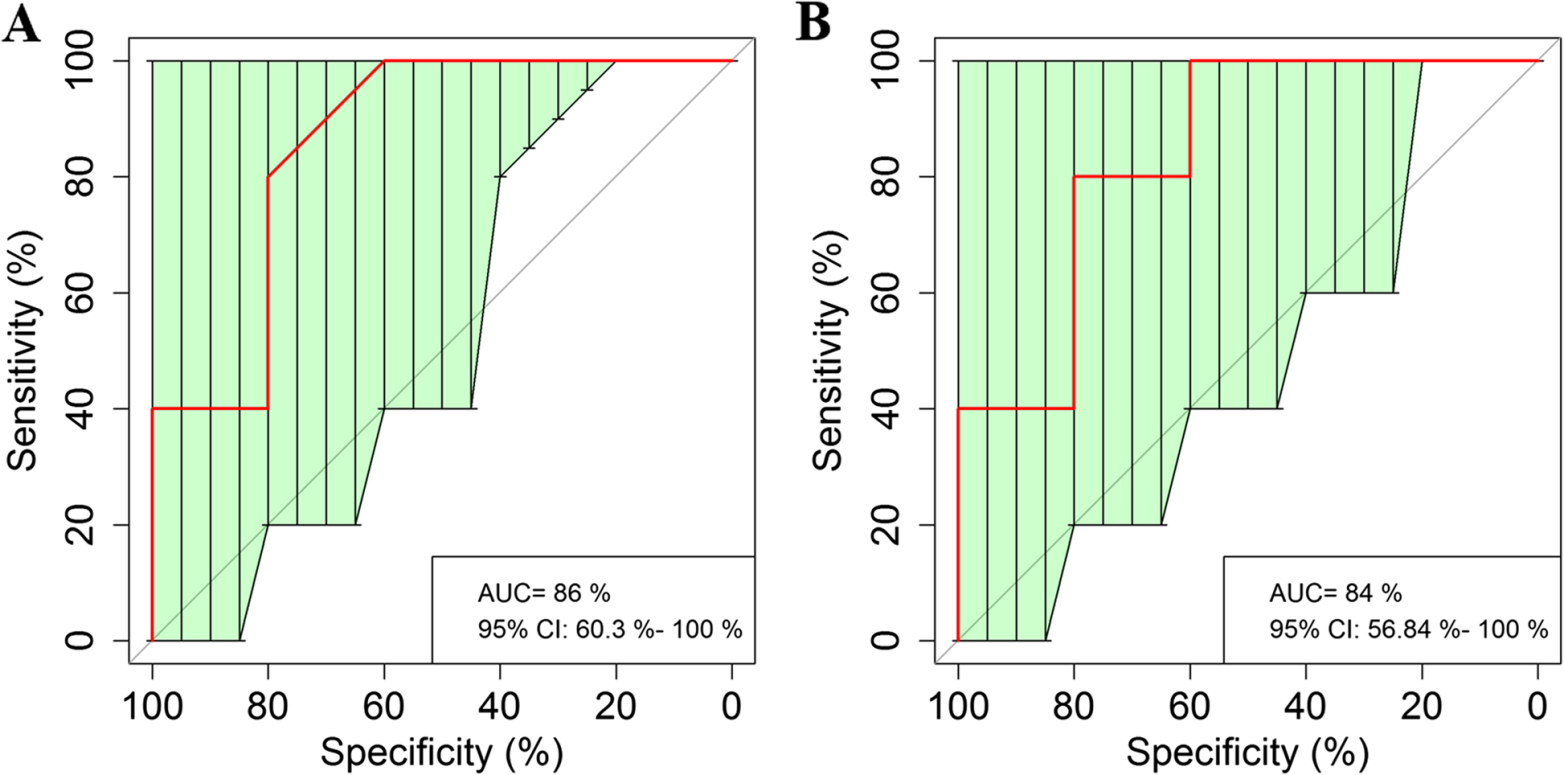
Receiver operating curve (ROC) for *Methanosarcina siciliae* and *Parasitella parasitica*. (**A**) *Methanosarcina siciliae*. The AUC was 86% with the 95% CI of 60.3 – 100%. (**B**) *Parasitella parasitica*. The AUC was 84% with the 95% CI of 56.84 – 100%

To identify the differences in gut fungi and archaea at the species level, we performed linear discriminant analysis (LDA) effect size (LEfSe) analysis on our metagenomic sequencing data. Results showed that four archaea species were significantly different in abundance across the three weaning periods (Fig. 3A), one of which was significantly elevated in the day 28 group and three of which were significantly elevated in the day 21 group. For example, *Methanosarcina siciliae* was enriched in the day 28 group, and *Methanolobus psychrophilus, Thermoplasmatales archaeon-*BRNA1*,* and *Thermoplasmata archaeon* were enriched in the day 21 group. On the other hand, only one fungi species, *Parasitella parasitica,* differed significantly in abundance across the three weaning periods, enriching in the day 28 group (Fig. 3B). A random forest analysis was then conducted to examine our ability to discriminate among the weaning periods based on fecal microbiota metagenomic sequencing of archaea (Fig. 3C) and fungi (Fig. 3D) species. As was found in LEfSe analysis, *Methanosarcina siciliae*, *Methanolobus psychrophilus,* and *Thermoplasmata archaeon* (archaea) and *Parasitella parasitica* (fungus) differed significantly among the three weaning periods and could therefore be used to distinguish among the three weaning periods with moderate to high diagnostic accuracy (area under the curve (AUC): 86% (Fig. 4A), 76% (Supplementary Fig. 3A), 60% (Supplementary Fig. 3B), and 84% (Fig. 4B), respectively).

### The archaea and eukaryotes associated with bacterial colonization and succession

**Fig. 5:**
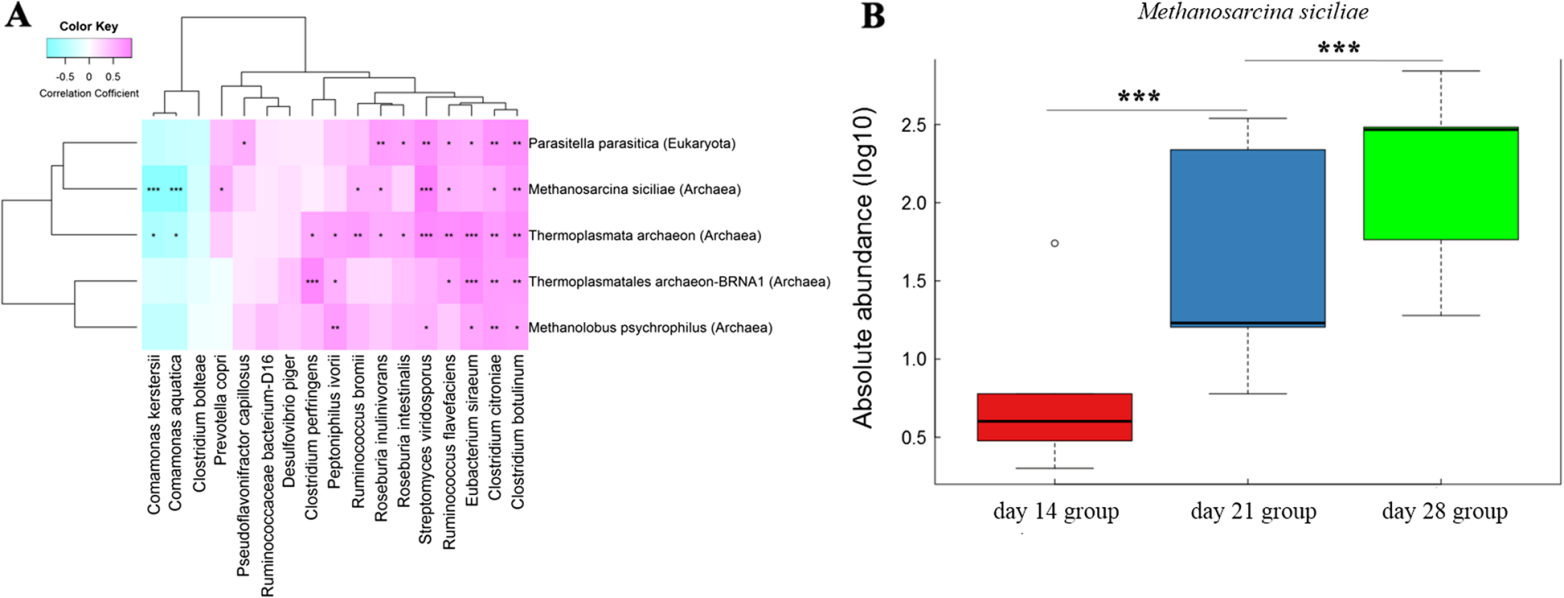
Levels of gut archaea and fungi species found to be significantly correlated with gut bacterial species, and the absolute abundances of *Methanosarcina siciliae*. (**A**) The heat maps showing the gut archaea and fungi species relationship with gut bacterial species. The *X*-axis represents the bacterial species. The *Y*-axis indicates the gut archaea and fungi species. * *P* < 0.05, ** *P* < 0.01, and *** *P* < 0.005. (**B**) The absolute abundances of *Methanosarcina siciliae* based on metagenomic sequencing results among the three weaning periods. * *P* < 0.05, ** *P* < 0.01, and *** *P* < 0.005

In our previous study, 17 bacterial species (e.g. *P. copri*), 8 KEGG functional terms and 21 CAZymes were identified to have significantly different abundances among the three weaning periods, and the abundance of bacteria *P. copri* played an important role in mitigating piglet adaptation during the weaning transition and was correlated with KEGG pathways and CAZymes [27]. Here, we used a Spearman correlation analysis to determine whether the archaea and eukaryotes are associated with bacterial colonization and succession, especially the bacteria *P. copri*. However, only one archaea (*Methanosarcina siciliae*) was found to be positively correlated with *P. copri* (Fig. 5A). The abundance of *Methanosarcina siciliae* was markedly higher in the day 28 group (*P* < 0.001, FDR) and lower in day 14 group (*P* < 0.001, FDR) compared to that in day 21 group (Fig. 5B). Furthermore, *Methanosarcina siciliae* abundance was positively correlated with most differential KEGG pathways except cationic antimicrobial peptide (CAMP) resistance (ko01503) pathway (Supplementary Fig. 4A). The sulfur relay system (ko04122) was positively correlated with all of the differential archaea and fungi species (Supplementary Fig. 4A). In terms of CAZymes enrichment, most of differential CAZymes were positively correlated with *Methanosarcina siciliae* abundance (Supplementary Fig. 4B), indicating the contribution of the archaea and fungi species to changes in CAZymes.

### Co-occurrence analysis among the gut bacteria, archaea, fungi and serum metabolites

**Fig. 6:**
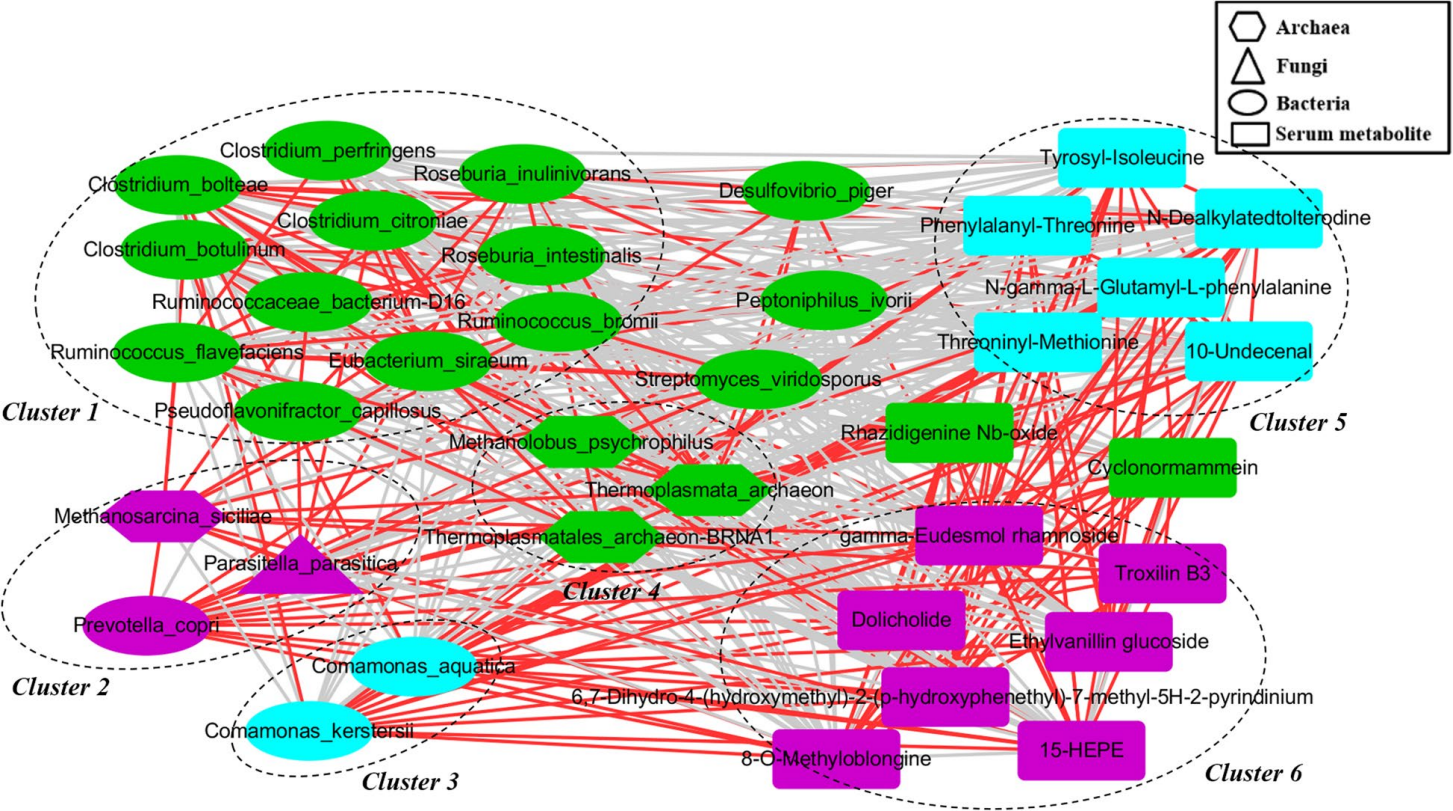
A co-occurrence network constructed from the relative abundances of differential bacterial species, archaea species, fungi species and serum metabolites. Purple, green and blue dots indicate the day 14 group, day 21 group and day 28 group, respectively. Edges between nodes indicate Spearman’s negative (light gray) or positive (light red) correlation

Previously, we identified a total of 15 metabolite features showing distinct enrichment patterns among the three weaning periods, including15-HEPE, 8-O-Methyloblongine, and Troxilin B3 that were enriched in the day 28 group [27]. Here, we explored potential correlations in abundances among these differential gut archaea, fungi, bacterial species and serum metabolites using a co-occurrence analysis. Bacterial and archaea species were found to form strong and broad co-occurring relationships with serum metabolites; fungi species, on the other hand, displayed only mild correlations with bacterial species, archaea species, and serum metabolites (Fig. 6). Within this coexpression network, bacterial species were clustered in two primary groups (cluster 1 and cluster 3). Serum metabolites also clustered into two groups (cluster 5 and cluster 6). Of note, the *P. copri*, *Methanosarcina siciliae* and *Parasitella parasitica* were positively correlated with each other’s, and found in cluster 2, while gut archaea species were mostly found in cluster 4. Furthermore, *P. copri* and *Methanosarcina siciliae* were positively and significantly correlated with 15-HEPE, 8-O-Methyloblongine, and Troxilin B3, indicating a synergistic and niche-related relationship.

## Discussion

Although gut fungi and archaea are essential member of the microbiota in animals, they have been neglected in the past years [28, 29]. Very limited number of previous studies have examined the composition of gut fungi and archaea. Of these, only a handful of studies have focused on how gut fungi and archaea communities develop and change during the piglet suckling and weaning periods [12, 24–26]. Here, we performed shotgun metagenomic sequencing to comprehensively characterize porcine gut fungi and archaea during the piglet weaning periods. Our results indicate that gut fungi and archaea communities are highly dynamic during suckling and weaning.

Consistent with previous studies, Ascomycota and Basidiomycota were the two most dominant phyla of fungi in pigs [30, 31], followed by Mucoromycota. Mucoromycota, characterized by the production of asexual aplanospores, fusion of gametangia to produce zygospores, and walls of chitin and chitosan [32, 33], is of interest because it is one of the few fungi interacting with bacteria. Fungal-bacteria interactions are the focus of a new field of microbiology with relevance to microbial ecology, host health and biotechnology [34]. For the archaea, Euryarchaeota was the most abundance phyla, followed by Methanobacteriaceae, Methanosarcinaceae, and Candidatus Methanomethylophilaceae. The significant role we found for Euryarcheaota in pig microbiomes suggests a previously underestimated role in human health and disease [35]. In addition, these sequences are similar to a number of clone sequences recovered from pig feces and cattle rumens [36], raising the possibility that a novel group of Euryarchaeota has adapted to live in the animal intestinal tract [35].

*Methanosarcina siciliae* and *Parasitella parasitica* were found to be the most significantly different archaea and fungi species among three weaning periods, respectively. Random forest analysis also identified *Methanosarcina siciliae* and *Parasitella parasitica* as a weaning-biased archaea and fungi at the archaea and fungi species level, respectively. *Methanosarcina siciliae* is known to be able to degrade dimethylsulfide and methane thiol to hydrogen sulfide, methane, and carbon dioxide [37, 38]. *Parasitella parasitica*, a facultative mycoparasite of zygomycetous fungi, forms cytoplasmic fusions with its hosts during infection [39], infecting many different mucoralean species from nearly all major genera [40, 41]. Moreover, *Methanosarcina siciliae* and *Parasitella parasitica* could be used to distinguish the three weaning periods with high diagnostic accuracy, suggesting that *Methanosarcina siciliae* and *Parasitella parasitica* are important species for mitigating piglet adaptation during the weaning transition.

The archaea were found to be ubiquitously present in the piglet gastrointestinal tract microbiome across the three weaning periods. In particular, *Methanosarcina siciliae*, the only differential archaea species, was significantly and positively correlated with the bacteria *P. copri*. A previous study focused on neonatal colonization found that archaea were transient and found almost exclusively only within the first few weeks of life [42]. Other studies suggest that archaea are core members of the adult gastrointestinal tract microbiome [43, 44]. Interesting, we also observed that KEGG pathways and CAZymes related to energy metabolism were positively associated with *Methanosarcina siciliae*. However, human archaeal commensal has a highly restricted energy metabolism [45], which makes it a specialized member of the gastrointestinal microbiome [44]. Furthermore, the *P. copri* and *Methanosarcina siciliae* were positively correlated with 15-HEPE, 8-O-Methyloblongine, and Troxilin B3. These serum metabolites may provide a mechanism for anti-inflammatory, and potentially act as a biomarker for food consumption and release of arachidonic acid and diacylglycerols [46, 47]. These results indicate that bacterial colonization and succession are associated with archaea and are thereby among the earliest colonizers of the piglet gastrointestinal tract microbiome.

## Conclusions

In this study, we found that gut fungi and archaea composition were significantly changed through time during the weaning processes. *Methanosarcina siciliae* and *Parasitella parasitica* were found to be the most significantly different archaea and fungi species, respectively, which were also identified by random forest analysis as weaning-biased archaea and fungi at the species level. *Methanosarcina siciliae* was significantly correlated with the changes of *P. copri* abundance, KEGG pathways and CAZymes. Furthermore, *Methanosarcina siciliae* and *P. copri* were positively and significantly correlated with 15-HEPE, 8-O-Methyloblongine, and Troxilin B3, indicating alterations of gut bacteria, archaea, fungi, and serum metabolites generated a characteristic weaning transition coexpression network. Overall, our results reveal important insights into the interactions among gut bacteria, archaea, fungi and serum metabolites and lead to beneficial understanding of gut bacterial colonization and succession associated with archaea.

## Materials and methods

### Experimental animals and sample collection

The gut microbiome of five Large White piglets (two males and three females) from three age strata was studied. All piglets stayed with their dams during the suckling period and were allowed to nurse freely until weaning began at 21 days old. Once weaned, piglets were transferred into the same pen and were provided with commercial formula diet and clean water ad libitum. According to previous studies [25, 48, 49], seven days before and after weaning day were chosen for other two periods. Fresh feces from all piglets were collected from each animal’s anus by rectal massage at 14 days of age (day 14 group), 21 days (the day of weaning, day 21 group), and 28 days of age (day 28 group). All piglets had no obvious disease or diarrhea and received no probiotic or antibiotic therapy during the period from birth to the end of this study. Fecal samples were immediately snap-frozen in liquid nitrogen for transportation and stored at − 80 °C for later use.

### Microbial DNA extraction

Microbial DNA was extracted from fecal samples using the QIAamp Fast DNA Stool Mini Kit (Qiagen, Germany) according to the manufacturer’s instructions. The concentration of DNA was determined using a Nanodrop-1000 (Thermo Scientific, USA) and the DNA purity was determined by 0.8% (w/v) agarose gel electrophoresis. All DNA samples were stored at -20 °C until further processing.

### Metagenomic sequencing analysis

Metagenomic sequencing of the 15 fecal microbial DNA samples was performed using a Novaseq-PE150 platform. Briefly, sequencing libraries were generated using NEB Next® Ultra™ DNA Library Prep Kit for Illumina (NEB, USA) by following manufacturer’s instructions, and index codes were added to attribute sequences to each sample. The clustering of the index-coded samples was performed on a cBot Cluster Generation System according to the manufacturer’s instructions. After cluster generation, the prepared libraries were sequenced on the Novaseq-PE150 platform and paired-end reads were generated. The raw data were preprocessed using Readfq in order to acquire clean data for subsequent analysis. As host pollution may exist in samples, the clean data was checked via blast against the host database using Bowtie2.2.4 software [50] to filter out reads that are of host origin. The clean data were then assembled and analyzed using SOAPdenovo software [51, 52]. The scaftigs were predicted the ORF using MetaGeneMark (V2.10) software, and length information shorter than 100 nt [51, 53] was filtered from the predicted results using the default parameters. For ORF predicted scaftigs, CD-HIT [54] software was used to reduce redundancy and create the unique initial gene catalogue. DIAMOND [55] software was used to blast the Unigenes with the bacterial, fungal, archaeal and viral sequences, all of which were extracted from the NCBI NR database. As each sequence may have multiple aligned results, we chose the aligned sequence result where the e value ≤ the smallest e value × 10 [56]. These sequences were used in the LCA algorithm which was applied to system classification of MEGAN [57] software to guarantee the species annotation information of sequences.

### Statistical analysis

Sparse Correlation for Compositional data (SparCC) was employed to determine co-abundance (positive) and co-exclusion (negative) relationships among differential gut fungi, archaea, bacterial species, and serum metabolites based on their relative abundances [58]. Network analysis was performed and visualized using Cytoscape (version 3.6.1). To identify the different abundances among groups, linear discriminant analysis (LDA) and effect size (LEfSe) analysis were performed under the condition α = 0.05, with an LDA score of at least 2.0 [58]. Story’s FDR was used to correct the multiple tests.

## Supplementary Information

**Additional file 1.**

## Data Availability

All of the data generated or analyzed during this study are available from the corresponding author on reasonable request.

## Abbreviations

AUC: Area under the curve
Bp: Base-pairs
CO_2_: Carbon dioxide
NCBI: National Center for Biotechnology Information
NR: Non-redundant
CAZy: Carbohydrate-Active enZYmes database
KEGG: Kyoto Encyclopedia of Genes and Genomes
SparCC: Sparse Correlations for Compositional
LDA: Linear discriminant analysis
LEfSe: Linear discriminant analysis effect size
