# Supplementary material for: Gut archaea associated with bacteria colonization and succession during piglet weaning transitions
Source: BMC Vet Res. 2022 Jun 24;18:243. doi: 10.1186/s12917-022-03330-4 (PMC9229118; doi:10.1186/s12917-022-03330-4)
Supplement: Supplementary file 1 — Additional file 1. [file 12917_2022_3330_MOESM1_ESM.docx]

**Supplementary Table 1 The sequence assembly analysis for shotgun metagenomic sequencing**

| **SampleID** | **Group** | **Total len.(bp)** | **Num.** | **Average len.(bp)** | **N50 Len.(bp)** | **N90 Len.(bp)** | **Max len.(bp)** |
| --- | --- | --- | --- | --- | --- | --- | --- |
| sample01 | day 14 | 264,156,618 | 137,189 | 1,925.49 | 3,370 | 689 | 311,606 |
| sample02 | day 14 | 260,171,306 | 133,047 | 1,955.48 | 3,746 | 686 | 340,330 |
| sample03 | day 14 | 195,592,579 | 83,442 | 2,344.05 | 5,024 | 774 | 254,666 |
| sample04 | day 14 | 197,707,695 | 89,314 | 2,213.62 | 5,439 | 713 | 592,496 |
| sample05 | day 14 | 184,559,247 | 99,310 | 1,858.42 | 3,321 | 663 | 452,237 |
| sample06 | day 21 | 291,761,609 | 148,095 | 1,970.10 | 4,142 | 682 | 286,734 |
| sample07 | day 21 | 253,358,141 | 117,510 | 2,156.06 | 4,826 | 713 | 761,807 |
| sample08 | day 21 | 263,634,987 | 132,116 | 1,995.48 | 3,993 | 694 | 722,641 |
| sample09 | day 21 | 256,646,172 | 124,006 | 2,069.63 | 4,712 | 693 | 382,918 |
| sample10 | day 21 | 239,910,236 | 116,367 | 2,061.67 | 4,390 | 705 | 308,611 |
| sample11 | day 28 | 154,430,111 | 79,172 | 1,950.56 | 3,690 | 685 | 465,557 |
| sample12 | day 28 | 193,422,424 | 87,987 | 2,198.31 | 4,782 | 733 | 768,060 |
| sample13 | day 28 | 172,716,624 | 69,192 | 2,496.19 | 8,609 | 760 | 393,425 |
| sample14 | day 28 | 197,818,435 | 101,366 | 1,951.53 | 3,483 | 698 | 457,626 |
| sample15 | day 28 | 106,318,122 | 48,047 | 2,212.79 | 5,458 | 722 | 380,442 |


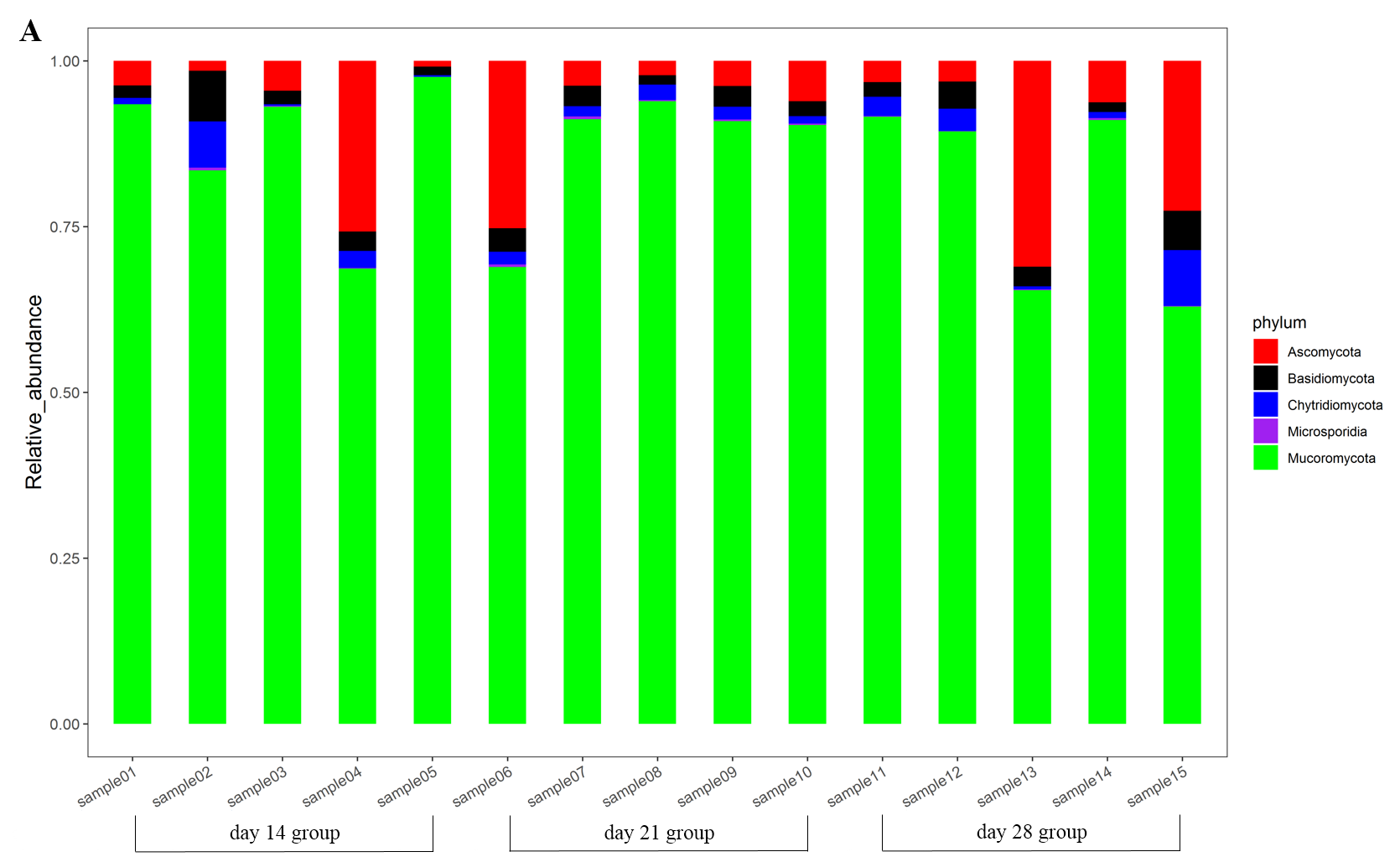


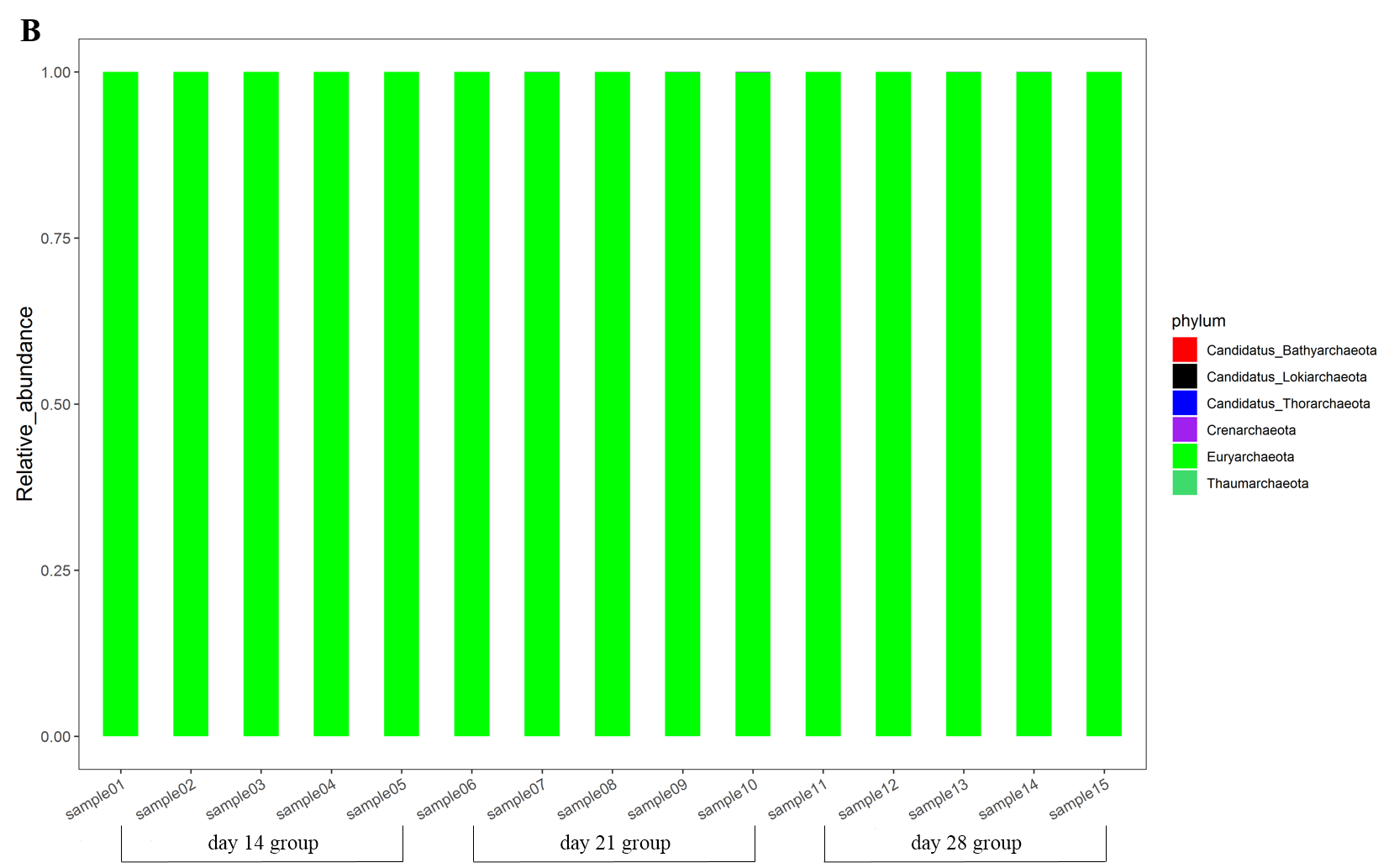


**Supplementary Figure 1** Shotgun metagenomic sequencing data for all tested samples. (A) Categories and relative abundance of gut fungi at the phyla level. (B) Categories and relative abundance of gut archaea at the phyla level.


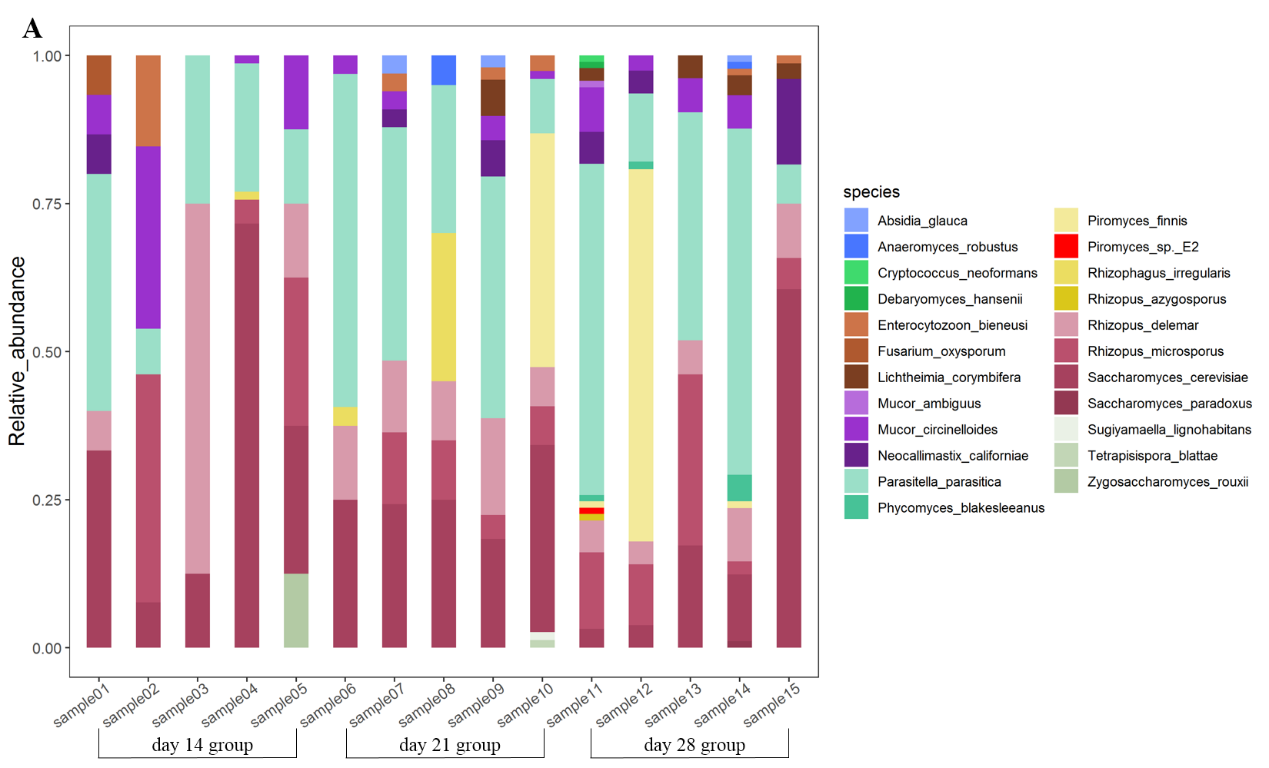


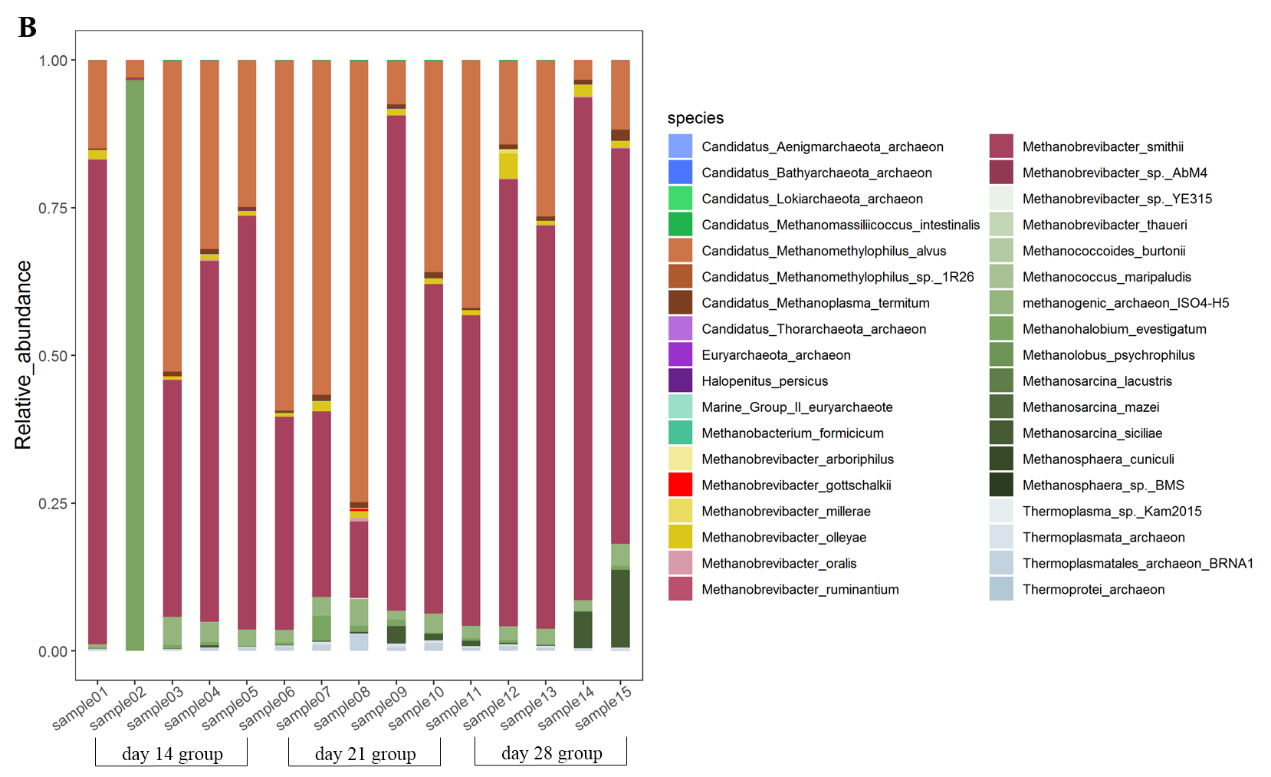


**Supplementary Figure 2** Shotgun metagenomic sequencing data for all tested samples. (A) Categories and relative abundance of gut fungi at the species level. (B) Categories and relative abundance of gut archaea at the species level.


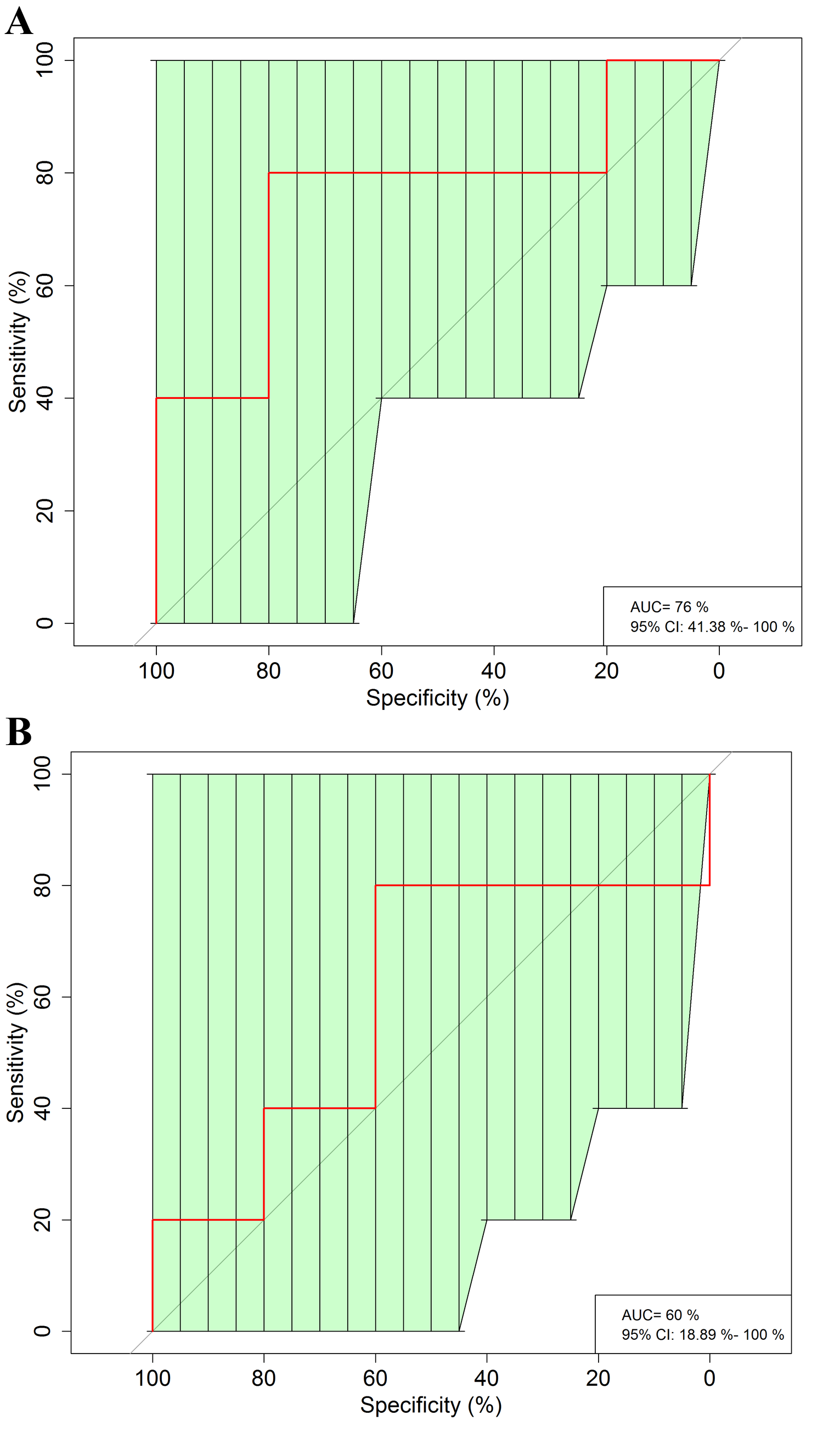


**Supplementary Figure 3** Receiver operating curve (ROC) for *Methanolobus psychrophilus* and *Thermoplasmata archaeon*. (A) *Methanolobus psychrophilus*. The AUC was 76% with the 95% CI of 41.38 – 100%. (B) *Thermoplasmata archaeon*. The AUC was 60% with the 95% CI of 18.89 – 100%.


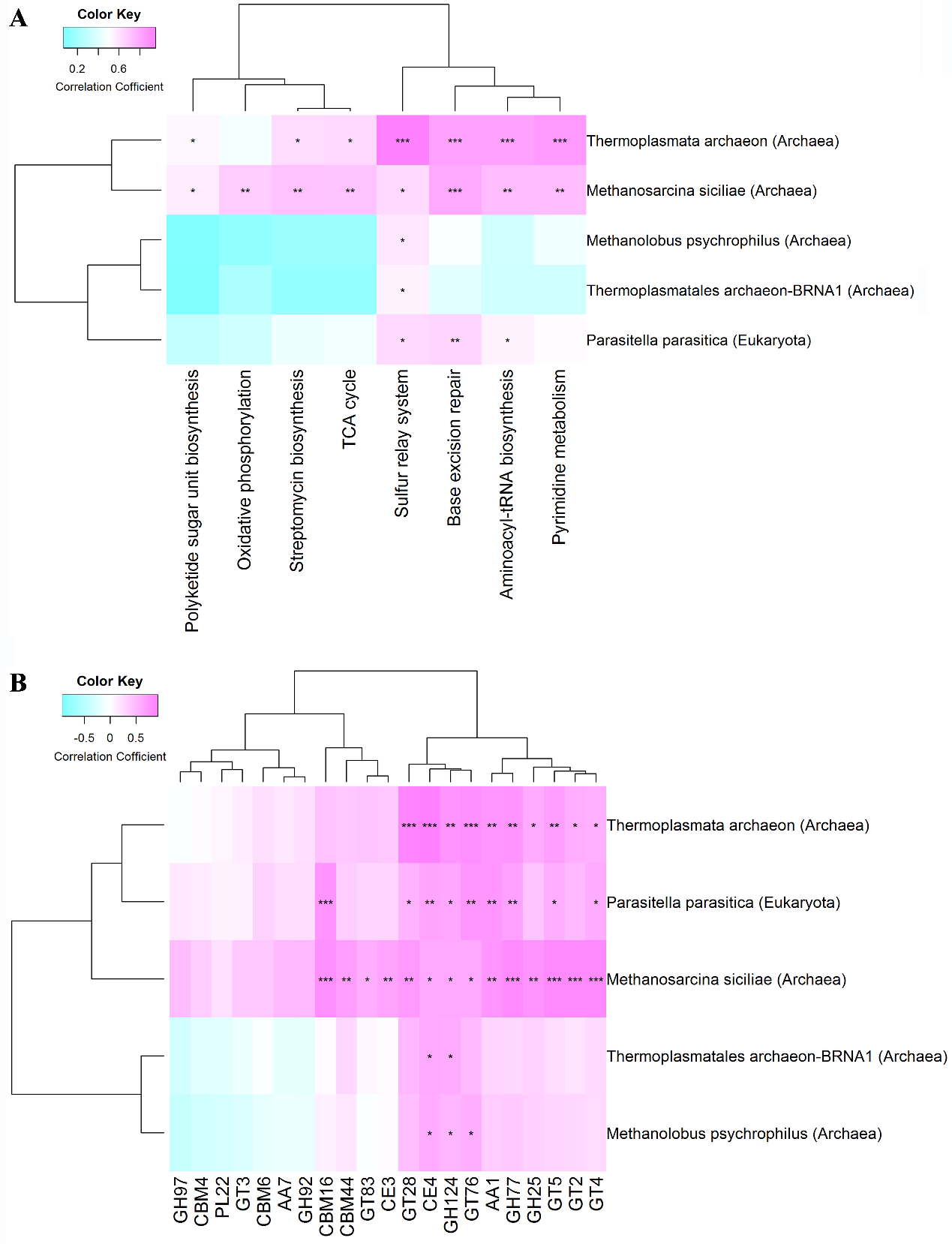


**Supplementary Figure 4** Levels of gut archaea and fungi species found to be significantly correlated with KEGG pathways and CAZymes. (A) The gut archaea and fungi species relationship with KEGG pathways. (B) The gut archaea and fungi species relationship with CAZymes. The x-axis depicts gut archaea and fungi species; the y-axis represents the differential gut bacterial species, KEGG pathways, or CAZymes.
